# Shelf-life, bioburden, water and oxygen permeability studies of laser welded SEBS/PP blended polymer

**DOI:** 10.1038/s41598-023-41477-8

**Published:** 2023-09-01

**Authors:** Satisvar Sundera Murthe, Srimala Sreekantan, Rabiatul Basria S. M. N. Mydin, Mugashini Vasudevan, Jimmy Nelson Appaturi

**Affiliations:** 1 School of Materials and Mineral Resources Engineering, Universiti Sains Malaysia, Engineering Campus, 14300 Nibong Tebal, Penang Malaysia; 2 Department of Biomedical Science, Advanced Medical and Dental Institute, Universiti Sains Malaysia, 13200 Kepala Batas, Penang Malaysia; 3 Department of Mechanical Engineering, Universiti Teknologi PETRONAS, 32610 Seri Iskandar, Perak Darul Ridzuan Malaysia; 4 De Eco SR Hygiene, Science and Engineering Research Centre (SERC), Engineering Campus, Universiti Sains Malaysia, 14300 Nibong Tebal, Penang Malaysia

**Keywords:** Chemistry, Engineering, Materials science

## Abstract

The most common material used for blood bags is PVC, which requires the addition of DEHP to increase its flexibility. DEHP is known to cross the polymer barrier and move into the stored blood and, ultimately, the patient's bloodstream. In this work, an alternative prototype composed of SEBS/PP was fabricated through blow-moulding and compared with the commercially available PVC-based blood bag which was designated as the control. The blow-moulded sample layers were welded together using CO_2_ lasers and optimized to obtain complete sealing of the sides. The samples' performance characteristics were analyzed using water permeability, oxygen permeability, shelf-life, and bioburden tests. The SEBS/PP sample exhibited the highest oxygen permeability rate of 1486.6 cc/m^2^/24 h after 40 days of ageing, indicating that the sample is conducive for red blood cell (RBC) respiration. On the other hand, the SEBS/PP sample showcased a lower water permeability rate of 0.098 g/h m^2^ after 40 days of aging, indicating a high-water barrier property and thus preventing water loss during storage. In comparison, the oxygen and water permeability rates of PVC-DEHP were found to be distinctly lower in performance (662.7 cc/m^2^/24 h and 0.221 g/h m^2^, respectively). In addition, shelf-life analyses revealed that after 40 days of ageing, polymer samples exhibited no visual damage or degradation. The optimal parameters to obtain adequate welding of the SEBS/PP were determined to be power of 60% (18 W), speed of 70 in/sec and 500 Pulse Per Inch (PPI). Furthermore, the bioburden estimates of SEBS/PP of 115 CFU are markedly lower compared to the bioburden estimate of PVC-DEHP of 213 CFU. The SEBS/PP prototype can potentially be an effective alternative to PVC-based blood bags, particularly for high-risk patients in order to reduce the likelihood of medical issues.

## Introduction

The inclusion of di-2-ethylhexyl phthalate (DEHP) as plasticizer in polyvinyl chloride (PVC) infusion tubing and blood storage devices is often touted as unavoidable due to its capability of imparting flexibility and durability as well as providing relatively good blood stability^1^. These characteristics of DEHP coupled with the nature of PVC of possessing good mechanical strength, low cost, ease of fabrication, clarity and ability to withstand centrifugation cements the suitability of plasticized PVC for blood storage devices^2^. This polymeric blend of PVC and DEHP confers the blood bag, weldability and thermal resistance during storage and steam sterilization^3^. Despite its significant benefits, individuals undergoing medical treatments such as blood transfusions, total parenteral nutrition, extracorporeal membrane oxygenation (ECMO) and haemodialysis are inevitably exposed to DEHP as a result of red blood cells (RBCs) coming into contact with plasticized PVC^3,4^. DEHP is lipophilic and not chemically bound to PVC and thus, readily migrates from the PVC polymer matrix into the lipid-rich stored blood upon contact^5,6^. This leaching effect poses a considerable danger to patients, particularly children and neonates, as DEHP is a well-known endocrine disruptor^7,8^. The DEHP plasticizer has toxic effects on the respiratory, immune and nervous systems and impairs renal, testicular, ovarian and neural functions^9^. DEHP has also been shown to induce stomatocytes and indirectly inhibit the ATP-dependent translocases that maintain the membrane's lipid asymmetry in RBCs^10^. Additionally, both the U.S. Environmental Protection Agency (EPA) and International Agency for Research on Cancer (IARC) have classified DEHP as a potential human carcinogen^7^.

However, current efforts to replace blood storage bags with alternative plasticizers have been unsuccessful and are primarily in the research and development phase^11,12^. Table 1 compares the significant research on blood bag materials and the drawbacks that plague current blood bags. These shortcomings of previous studies provide an opportunity to fabricate an effective and commercially viable PVC-free blood bag. There are several alternatives available currently such as ethylene vinyl acetate (EVA), polyolefins [i.e., polyethylene (PE), polypropylene (PP)], polyurethanes (PU), fluoropolymers and thermoplastic elastomers [i.e. styrene-ethylene-butylene-styrene (SEBS)]^13^. However, the limitations, such as the non-steam sterilizable nature of PU, and the non-sealable or weldable characteristic of polyolefins, hinder its widespread use^13^. Furthermore, alternative attempts to remove DEHP, have failed to achieve acceptable mechanical properties comparable to conventional blood bags. Alternative works are also unable to achieve a high tensile strength while maintaining a low Young's modulus^14,15^. One prior study replaced DEHP with alternative plasticisers that are expensive, foul-smelling or materials whose long-term effects on human health are not known^11^. The alternative polymers are not able to be steam sterilised, thereby increasing the cost of sterilisation while other alternative polymers are also unable to withstand the required cold storage of up to −40 °C, which prevents the storage of rare blood^11^.

**Table 1:** Comparison of major studies on alternative blood bag materials and the drawbacks.

Polymers	Polymer blend composition	Mechanical properties	Plasticizer	Compatibilizers/ Additives	Steam Sterilisation	Comments	References
Thermoplastic polyurethane and polypropylene (TPU/PP)	50/50, 70/30, 80/20	Tensile strength of 3.59, 13.20, 18.23 MPa, respectively	None	N/A	N/A	The Young's modulus of the polymer blend compositions drastically deviates from the Young's modulus of the blood bag	^14^
Thermoplastic polyurethane, polypropylene and ethylene vinyl acetate (TPU/PP/EVA)	50/50, 70/30, 80/20, 80/20/5	Tensile strength of 3.59, 13.20, 18.23, 14.21 MPa, respectively	None	EVA	Yes	The Young's modulus of the polymer blend compositions drastically deviates from the Young's modulus of the blood bag. The polymer blend is biocompatible	^15^
PVC	N/A	N/A	Tri-(2-ethylhexyl) trimellitate (TOTM)	N/A	Yes	As the migration of the plasticiser is less than that of DEHP, the stable RBC shelf life is only 21 days	^33^
PL-732—polyolefin (polypropylene and a thermoplastic elastomer ethylene butylene copolymer and polystyrene)	N/A	N/A	None	Hindered phenolic antioxidant, specifically 1,3,*S*-trimethyl2,4,6-tris (3,*S*-di-tert-butyl-4-hydroxybenzl) benzene (BHBB)	N/A	PL-732 is neither weldable nor sealable by high frequency due to its nature as a non-polar polymer. A high content of the hindered phenolic antioxidant can be found in the blood bag	^34^
PVC	N/A	N/A	Butyryl-tri-*n*-hexyl-citrate (BTHC)	N/A	No	The plasticiser releases an unpleasant odour, has an elevated price and the lack steam sterilisation capabilities have restricted its use	^11^
PVC	N/A	N/A	Diisononylester of cyclohexane dicarboxylic acid (DINCH)	N/A	N/A	Although PVC-DINCH produces a haemolysis level after 42 days of storage similar to DEHP-PVC with a lower toxicological effect, the plasticiser migrates into the blood nevertheless and the long-term effects of DINCH on health is not yet well studied	^35^
Polyolefin/2-methacryloyloxyethyl phosphorylcholine (MPC)—hydroxyethyl methacrylate (HEMA)—lauryl methacrylate (LMA) copolymers	MPC:HEMA:LMA = 1:3:6	Tensile strength of 8.77 MPa	None	N/A	N/A	The MPC copolymer synthesis requires a complex multistep process which may contribute to higher production costs. An alternate study has also shown that steam sterilisation and other means of sterilisation affects MPC to varying degrees	^1,36^

Compared to the available blood bag alternatives, SEBS/PP can withstand steam sterilisation and cold storage^16–19^. The preliminary material selection for this study was based on our previous work, wherein SEBS/PP exhibited approximate or improved physical characteristics to PVC-DEHP on parameters such as hardness, flexibility, strength and thermal stability^20^. SEBS polymers are high-impact polymers with high elongation at break, low processing temperature, low melt viscosity, and low distortion during extrusion, which allows them to be utilised for a myriad of applications^21^. Its uniqueness lies in the fact that it has the processability of thermoplastics and the elasticity of rubber without having to undergo vulcanisation^22^. However, SEBS has poor mechanical strength and toughness, which prevents it from being utilised comprehensively and has been mostly relegated to use as an impact modifier. Fortunately, PP polymers are tough and strong polymers that can be utilised to complement SEBS while leveraging the benefits of both materials. Due to its highly versatile nature and low cost, PP has been employed in many scientific, domestic, and industrial applications. Studies have shown that the combination of SEBS and PP is rational as physical blending occurs conveniently due to the similar chemical makeup of PP and the ethylene-co-butylene (EB) midblock of SEBS^23^.

However, despite the availability of various physical, chemical and thermal characterisations of SEBS/PP based materials and other alternative materials for blood bag, no significant study has been conducted to properly evaluate the performance properties of these materials as a prototype aside from extensive biocompatibility and haemocompatibility studies. The selection of alternative PVC-free materials for use as blood storage containers must replicate to a high degree the essential physical properties of commercial PVC blood bags such as good visual transparency post-processing, high oxygen and low water permeability, weldability, leak resistant and long shelf life^24,25^. As radio frequency (RF) welding works best on PVC-based materials and does not provide effective welding in poor dipole materials such as polypropylene it is necessary to explore other means of welding for alternative materials in order to form viable blood bags^26^. The welding of polymers by laser welding eliminates the need for adhesives which has associated risks such as prone to leaching, short-term stability and slow processing^27^. Nevertheless, the transparent nature of SEBS/PP may pose a problem to laser welding as laser welding most commonly requires a non-transparent, absorbing layer.

Ruotsalainen et al. (2015) demonstrated that it is possible to join two transparent polymers despite the thin nature of the polymers through the quasi-simultaneous laser scanning method and varying the wavelength of the laser^28^. Jansson et al. (2005) showcased that quasi-simultaneous laser welding can be successfully utilised for welding plastics during mass production^29^. This can be highly advantageous if utilised for the prototyping of SEBS/PP polymer which is intended as an alternative to the commercial PVC-DEHP blood bags. Furthermore, Nguyen et al. (2020) showed that the semi-crystalline polypropylene which is not suitable for RF welding, can be readily laser welded through the use of beam sources that emits radiation within the polymeric intrinsic absorption bands between 1500 and 2000 nm^30^. The drawback of large heat affected zone was overcame by using quasi-simultaneous laser scanning. These studies indicate that the multi-pass quasi-simultaneous laser scanning is highly suitable technique for the fabrication of SEBS/PP polymer. However, most of the studies in the available literature laser welds the polymers by employing optimal or adequate clamping force and no comprehensive attempt is yet made to weld polymers by using little to no clamping force whilst varying the welding parameters during process optimisation, in particular for multi-pass welding variant of the laser transmission welding process. On the other hand, the studies on performance characteristics mostly focuses on oxygen permeability such as the study by Murata et al. (2021) that evaluated the effects of polyolefin bags on stored blood which attributes the improved platelet storage to a higher gas permeability^31^. Other performance studies does not focus on the performance of the alternative materials but the effects components of the blood bag such as the work of Serrano et al. (2010) that evaluates the effects of bag in-line closure device and its shear inducing capability in blood^32^. Critical study on the nature of the performance characteristics is lacking as it mostly focusses on the biological effects resulting from these parameters as opposed to studying the properties of the alternative materials.

In this study, a multi-pass laser transmission welding of SEBS/PP is explored whilst exerting minimal clamping pressure on the layers in order assess the weld strength of the material. A CO_2_ laser was employed in this to weld the layers of polymers to fabricate rudimentary blood bag prototypes. The aim of this study was to understand whether the SEBS/PP polymer blend would be able to be laser welded in order to form a blood container and assess its weld strength, weld quality and visual appearance. Additionally, the study seeks to assess whether the laser welded polymer device is capable of emulating or improving upon the performance properties of commercial PVC-DEHP blood bags. The study on the performance characteristics was conducted to ensure that the SEBS/PP material is capable of storing blood and its components at par or better than commercial PVC-DEHP blood bags. In order to assess the SEBS/PP blood bag material and its laser weldability, the edges of the material were laser welded and the welded blood bag prototype's performance characteristics and efficiency were investigated for oxygen and water transmission rate, bioburden and shelf life.

## Methodology

### Materials

The thermoplastic elastomer, styrene-ethylene-butylene-styrene (SEBS), G1645V with a polystyrene content of 11.5% to 13.5% and Shore A hardness of 35 was acquired from Kraton Polymer (Wesseling, Germany). The SEBS has an enhanced midblock (ethylene-butylene), which increases compatibility for blending with polypropylene. The polyolefin, polypropylene random-heterophasic copolymer (PP), Bormed SC820CF-11 with a tensile strength of 50 MPa and melt flow rate of 3.9 g/10 min. The PP was procured from Borealis AG (Vienna, Austria). All polymeric materials were used after drying the polymer pellets sufficiently to remove moisture so as not to affect the quality of the blow-moulded material. The polymers were of medical grade, suitable for blow-moulding and were processed without any plasticizer, additives or thermal substrates. The PVC blood bag samples with an unknown percentage of DEHP and processing additives were obtained from Terumo Penpol Limited (Kerala, India). The tubes of the samples were cut and all preservative solutions were drained. The blood bags were washed once with 70% ethanol and distilled water twice to remove any adhered impurities. In this study, two different sets of storage containers were used. The first set comprises SEBS/PP blow-moulded film devoid of processing substrates and plasticizers. The second set consists of the procured commercial PVC blood bag used as it is without any further chemical modification to the inner or outer surface of the blood bags except for the stripping of any attached preservative solutions. Handling all sterilised samples and their corresponding films in an aseptic manner was essential to prevent contamination.

### Preparation and blow-moulding of SEBS/PP

The pre-dried SEBS and PP polymer pellets were carefully weighed and measured based on a minor modification of our previous work Murthe et al.^20^. Briefly, the SEBS and PP polymers were mechanically and manually mixed in the optimal composition of 57.5 and 42.5 wt%, respectively. The mixture of SEBS and PP pellets was then fed into the single-screw extruder (Queen's Machinery Co. Ltd., Taipei, Taiwan) for free-blown film extrusion vertically upwards with a screw diameter of 30 mm and a screw length of 600 mm. The temperature was set to 190 °C in the feeding zone and 210 °C in the metering zone. The barrel in the feed section of the plasticating extruder system was devoid of grooves and entirely relied on the screw extrusion system for mixing. The extruder was equipped with a 2.6 kW direct current motor. The throughput was kept constant throughout the process, and the cooling system was kept constant after optimization to ensure good bubble stability, drawability, and a minimal film thickness deviation. The crosshead die for film blowing was of typical construction. In the die, there was a core of 50 mm in diameter with an opening through which the air blew the film. Around the core was a ring gap for film extrusion; beyond it, there was a gap for cooling the extruded film. The air blowing and cooling for the film came from a 0.55 kW compressor. The blown, cooled and flattened film was pulled through a set of nip rolls and then rolled up on the winder. The thickness of the films was then measured to ensure uniformity and then cut in batches.

### Welding of blow-moulded SEBS/PP

The weldability of the blow-moulded SEBS/PP film and the optimal set of parameters to seal the sides of the films to form a viable storage prototype is conducted by polymer welding studies. The blow-moulded SEBS/PP polymer films were welded using a laser-welding method. The polymer films were washed thoroughly twice using deionized (DI) water and flattened to the platform. A CO_2_ laser (λ = 680 nm) engraving system (V-460, Universal Laser System, Scottsdale, Arizona, USA) was used to weld the films on three edges. Cellophane tap, notoriously transparent to laser radiation, was used to grasp the samples during the welding process. The samples were preliminarily positioned inside the masks. The system was clamped with special clips to impart adequate pressure in the welding area and facilitate the welding between the two SEBS/PP films. At the same time, this gripping system prevents air entry into the overlapping area of the edges of the two samples. Focusing has always occurred at the interface surface between the two samples to be coupled. The varied parameters in the CO_2_ laser were power, speed, and the number of scans.

Additionally, the number of laser scans was also varied. The power limits the intensity of the laser that penetrates the SEBS/PP film, whereas the speed controls the rate at which the nozzle travels in the X–Y direction. The Pulse Per Inch (PPI) determines the number of pulses with which the laser beam hits the SEBS/PP film per inch. The varied parameters of the CO_2_ laser in this study are provided in Table 2 below. The laser-scribing process was conducted in raster mode and performed under ambient conditions. Based on the author's experience, the set of parameters is described in Table 2. The parameters were chosen based on an iterative process based on the immediate observations after welding with the previous set. The parameters were varied and tested until polymer welding reached optimal levels to study its weldability.

**Table 2:** Power, speed and pulse per inch of CO_2_ laser welding parameters used in this investigation.

Sample number	Power (%)/W	Speed (in/s)	Pulse per inch	Thickness (mm)	Number of Scans
1	50/15	50	500	~ 0.4	1 (one side)
2	50/15	50	500		2 (one side)
3	50/15	50	500		3 (both sides)
4	80/24	50	500		1 (one side)
5	85/25.5	50	500		1 (one side)
6	100/30	50	500		1 (one side)
7	80/24	70	500		1 (one side)
8	60/18	70	500		3 (one side)

### Oxygen permeability test

The oxygen permeability of the polymers in this study was determined following ASTM D3985-17 (Oxygen Gas Transmission Rate through Plastic Film and Sheeting Using a Coulometric Sensor) using an Oxygen Permeation Analyzer (ILLINOIS Instruments 8501) in a dry test environment with relative humidity less than 1%. All tests in the chamber were performed at 23 °C normalized to 1 atm partial pressure difference. The commercial PVC-DEHP blood bag and SEBS/PP samples were examined before and after accelerated ageing (20 and 40 days). Three replicates were conducted for each series of tests. The test specimens were cut and trimmed to a size suitable for the diffusion cell in which it will be mounted. The samples were then mounted as a sealed semi-barrier between two chambers at ambient atmospheric pressure. Purified gas was used as the test gas for both the samples. A stream slowly purged one nitrogen chamber while the other chamber contained the oxygen. As the oxygen gas permeated through the sample into the nitrogen carrier gas, the mixture was transported to the coulometric detector where it produced an electrical current, the magnitude of which is proportional to the amount of oxygen flowing into the detector per unit time. The polymer samples were thoroughly cleaned before being placed in the test chamber to prevent measurement inaccuracies. The oxygen transmission study was conducted for 24 h.

### Water permeability test

Water vapor permeation into the stored blood can damage the red blood cells. A water transmission rate study was conducted to determine the water permeability of the commercial and blow-moulded samples. The permeation of water vapour was measured following ASTM E96 – 95 (Standard Test Methods for Water Vapour Transmission of Materials) using the water method. All the tests in the chamber were performed with distilled water at 25 °C in a 50% humidity environment for 4 days. The commercial PVC-DEHP blood bag and SEBS/PP samples were tested before accelerated aging and 20 and 40 days after accelerated aging. Three replicates were conducted for each series of tests. Briefly, a metal cup was filled with distilled water while allowing for generous air space to reduce the possibility of water touching the polymer sample. The specimen is then attached to the cup and sealed. The cup and test polymer assembly were then weighed and placed in the test chamber. The test sample was periodically removed every 24 h and weighed. The weight loss as a function of time was recorded. All weight losses with both polymer specimens were therefore attributed to the permeation of water vapour. The water vapour transmission rate was calculated using the formula below:

$$WVTR=\frac{Wi-Wf}{T\times A}$$

where, WVTR is the water vapour transmission, g/h m^2^; W_i_ is the initial weight of gravimetric cup with sample and water (Day 0), g; W_f_ is the final weight of gravimetric cup with sample and water (Day 4), g; T is the period from W_i_ (Day 0) to W_f_ (Day 4) in hours; A is the surface area of the tested sample (open cup surface area, π (0.03)^2^, 2.8286 × 10^–3^ m^2^.

### Shelf-life test

The service life of blood bags is usually measured in years and testing the polymer weathering in real-time conditions over several years is inherently unrealistic or impossible. Additionally, both PVC-DEHP and SEBS/PP polymers are highly time and temperature-dependent due to their viscoelastic nature^37^. In light of this factor, accelerated ageing was employed to ascertain the useful lifespan of the test samples. This test suits relatively new products that have not yet gone through their useful lifespan. Accelerated aging is essentially conducted by simulating the period claimed for product expiration. The shelf-life was performed according to ASTM F1980-21 using accelerated aging parameters by Arrhenius’s equation method. Simulation is achieved by varying temperature and humidity levels and thus exposing the packaged device to conditions it is expected to experience during its shelf life. The test specimens were stored for 40 days to simulate a target shelf life of 365 days. The accelerated aging temperature (TAA) was set at 55 °C, the real-time ambient shelf temperature (TRT) was set at 23 °C and the relative humidity was kept constant at 45%. The Q10 (reaction rate factor) was 2 and the accelerated aging factor (AAF) was 9.19. Arrhenius’s equation is provided below:

$$Accelerated \, Aging \, Time \left(AAT\right)=\frac{Desired \, Real\, Time (RT)}{Q10 [\frac{TAA-TRT}{10}]}$$

### Bioburden test

Bioburden is the total number of viable contaminant microbes (bacteria, yeast and mould) on medical devices prior to sterilization. The initial bioburden on medical devices significantly affects the final sterilisation parameters of medical devices^38^. Therefore, the bioburden test was conducted to measure the initial bioburden of SEBS/PP and PVC-DEHP samples before use. The method for bioburden quantification of all surfaces was based on ISO 11737-1 (2018) using the membrane filtration technique. Briefly, ten samples of SEBS/PP and PVC-DEHP each were treated to remove microbes from tissues by sonicating the test samples without glass beads in 40 ml sterile saline solution for 10 min^38,39^. The prepared extract was manually shaken and allowed to pass through a membrane filter (nitrocellulose membrane) of pore size 0.45 µm. For incubation, the membrane filters were directly placed on Soybean–Casein Digest Agar (SCDA) surface to produce visible colonies. The incubation conditions were set at 30 ± 5 ºC for 6 days. Colonies produced on the surface of the membrane filter were counted and expressed as CFU/ml (colony-forming units). The bioburden estimate was calculated from a value obtained for the number of microorganisms comprising the bioburden by applying to the viable count, a correction factor compensating for the recovery efficiency. The correction factor and bioburden estimate were calculated as follows:

$$Correction\, Factor = \frac{100}{Average \, recovery \, by \, 1st\, treatment}$$

$$Bioburden \, estimate =Correction \, factor\, \times \, Average\, total \, recovery \, number (CFU)$$

All treatments utilized during bioburden estimation were handled aseptically and avoided conditions that affected the viability of microbes.

### Statistical analysis

The experiments were carried out in triplicates for oxygen and water permeability tests, and in replicates of ten for bioburden tests. The statistics were analysed using t-test in the Excel software for Windows (version 2019, Microsoft Corporation, USA). All the data were presented as mean ± standard deviation. The results were considered significant when the p-value is < 0.05.

## Results and discussion

### Weldability

**Figure 1:**
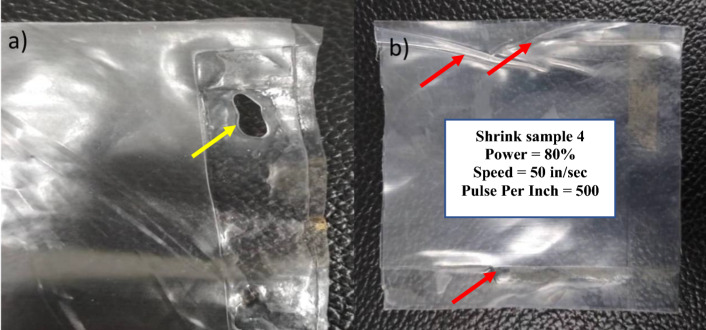
Laser welded samples with defects of (**a**) yellow arrow shows the holes and (**b**) red arrows show wrinkling, shrinkage and air pockets (for interpretation of the references to colour in this figure legend, the reader is referred to the web version of this article).

The welding optimization permits the study of a sizeable range of operational processing windows through visual inspection, thereby determining the parameters that lead to good welded joints. The welding between the polymer layers occurred through transmission welding, wherein the polymer sandwich's base material absorbs the laser's heating and melts. Essentially, the transmitting layer allows the laser beam to penetrate and the base layer absorbs the laser radiation to generate heat, which melts both polymer layers to fuse them^40–42^. The initial welding parameters in set 1 were set with a power of 50 as the minimum power required to heat and melt the layer of polymer adequately was found to be 50 based on prior experiences. The parameters in set 1 lacked any form of welding as it did not have the adequate power required to weld the joints. An approximately similar parameter with the laser being scan over the top layer twice was conducted in set 2 to analyze the effects of low power and multiple scans. The sample exhibited a slightly heated surface at the joint interface between the two layers. The exact parameters were repeated in sample 3 with the slight modification that each side of the polymer film sandwich was lasered three times each. However, while the welding regions of the polymer films were slightly glossy due to the polymers being molten on the contact surface, the films in set 3 were not welded to each other. This can be attributed to the low laser power indicating a higher temperature is required to reach the melting point of the polymers. Sets 4 and 5 were put through a higher laser power of 80 and 85 respectively while the speed was kept at a constant 50. Both samples showcased shrinkage and wrinkling of the polymers as well as the presence of air pockets. Interestingly, the edges of the samples were weakly attached and welded, although it can be easily separated by meagre force. Figure 1 below shows the laser-welded samples that exhibit defects in their structure.

**Figure 2:**
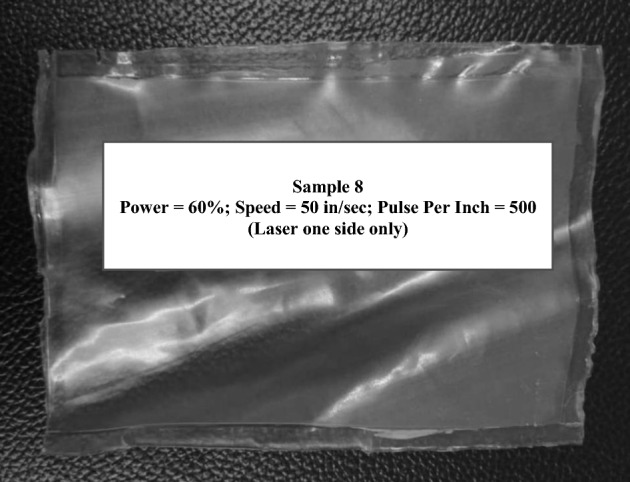
Optimized laser-welded sample with no visible defects.

In an attempt to solidly weld the entire portion of the polymer film, the power of the CO_2_ laser was increased to 100. The high power of the laser did not weld the polymer but created holes and burnt melted through the top layer of the film sandwich that was exposed to the laser beam. The parameters for set 7 was selected on the basis that sets 4 and 5 exhibited the most reasonable outcome out of the parameters studies thus far. The laser's speed was increased to 70 to account for the use of high power. Despite the higher speed, the sample was damaged and showed the presence of holes. Therefore, the laser beam power was reduced to 60 while maintaining the speed at 70 for the welding of set 8. Set 8 alone showcased acceptable joint aesthetics with no evidence of shrinkage, wrinkling, air pockets or gaping holes in the area scanned by the laser beam. The samples' layers adhered firmly, unlike sets 4 and 5. The entirety of lasered region was well sealed through welding. This optimized parameter can be attributed to several factors. The laser power of 60 possessed the adequate amount of power necessary to melt and weld the polymers without deteriorating the structure of the polymers. This factor coupled with the high speed of the laser which prevents the laser concentrating at a single position for too long thereby leading to degradation. However, the polymer has to be lasered 3 times on a single side to achieve optimal welding. At optimized parameters, the polymer film indicates a relatively higher absorptivity of laser radiation than the other sets of parameters, thus permitting the melting of the polymeric substance without adding any light-absorbing additives^40^. Figure 2 shows the optimally laser-welded blow-moulded film that is free of defects.

However, as a whole, the topmost transparent SEBS/PP polymer film cannot effectively absorb the heat, necessitating a high number of scans and flipping of the irradiated surface^43^. In addition, as a non-excessive force was applied to clamp down the polymer sandwich, the force applied on the sample, the molten material is pressed out of the joining zone^44^. Therefore, no excess material is pushed to the surfaces to create bead-like material on the surface. Parameters beyond the optimized range would mean working with laser radiation transmitted towards the surface that is too low or high, leading to ineffective welding or potentially to polymer degradation^43^.

The optimisation process in this welding study can be divided into three distinct divisions. The first division is that of Samples 1 to 3, in which the samples were welded using a power of 15 W whilst varying the number of scans. The number of scans was assessed to determine whether multiple lasering of the same polymer regions shows improved polymer welding. As elucidated earlier, the Samples 1 to 3 was not able to be stably welded. This can be attributed to the low laser power irradiated on the samples which only supplies low energy to the weld, thereby leading to insufficient material fusing^45^. In addition to that, due to the low power, the laser was not able to sufficiently penetrate the depth of the polymer. Therefore, at low laser beam powers, a stably welded joint cannot form, and the joint resistance is consequently zero^43,46^. In this instance, the laser radiated on the joint interface could not initiate the polymer's melting at the interface. Therefore, the joint interface between the two materials does not weld together completely or a weakly formed weld structure is formed as in this study. As such, no significant welding was observed in Samples 1 to 3 even though multiple number of laser scans were conducted on the SEBS/PP polymer.

The second division is that of Samples 4 to 6. In this division, the power of the laser was gradually increased while all other parameters were kept constant. This optimisation was conducted as previous studies have shown that increasing the laser power leads to a higher energy being supplied and thereby increases the heat input resulting to optimal material interfusion^45^. However, it can be visibly noticed that with higher laser power, the ablation of SEBS/PP polymer takes and which disrupts the joint welding, thereby introducing defects such as shrinkages and holes. This can be attributed to the fact that a high laser power leads to a high heat input that crosses the decomposition temperature of SEBS/PP polymer^47,48^. The weld strength of the polymer is decreased and defects are introduced into the polymer samples as a result of this. The high laser power may also increase the penetration depth in which the laser may penetrate through the welded region thereby degrading the structure of the weld. Research has shown that high laser power is capable of high depths of penetration in both the transparent and absorbing layer^49^. This can be attributed to the high energy deposition and heat diffusion into the SEBS/PP polymer by the high-power density and relatively lower irradiation time. Additionally, the relatively lower scanning speed provides increased laser interaction time with the polymer, thereby imposing a higher line energy^48^. This in turn results in overheating, burning and degradation of the polymer (partial or complete) and consequentially forms a weaker polymer weld strength^47,48^. Moreover, when the laser beam power is relatively high, the degradation of the polymeric material occurs and when this is coupled with high melt volume, this leads to defects and deters the formation of proper welds^46,50^.

This can be attributed to the violent and rapid melting of SEBS/PP owing to excess radiation on the joint's surface. The excessive heat that naturally accompanies the excess radiation, the vapourised polymer at the interface tends to form large gas bubbles at the interface, which in turn forms the air pockets. The formation of air bubbles largely prevents the welding of the films, resulting in insufficient sealing. Additionally, increasing the power of the laser increases the heat input directed toward the material and reduces the viscosity of the molten polymer. This characteristic indicates that the polymer is softened with the increase of time^44^. Furthermore, setting the laser beam with high power leads to erstwhile shrinkage or wrinkling^43^. The rapid heating and cooling due to the thermal expansion of the polymer film surface in the molten state when irradiated and the rapid contraction following its cooling after the laser beam has passed thereof leads to surface wrinkling or shrinkage. This condition is not readily visible under low laser power and speed as the thermal transitions are relatively slower and permit a longer time for the material to adapt to the internal stresses. These aforementioned results implies that the weld strength is highly influenced by the laser power in which very high laser power leads to polymer decomposition whereas very low laser power leads to insufficient or poor welding.

The third division is that of Samples 7 and 8. In this division, the power of the laser was lower than Samples 4 to 6 but higher than Samples 1 to 3. The scanning speed was also increased in this division to reduce the contact time between the medium laser power and polymer surface. Moreover, Sample 8 was scanned 3 times in order to study the welding of the polymer at multiple scans of medium power. This optimisation was conducted by considering studies have showcased that increasing the scanning speed leads to the reduction of laser interaction time^45,49^. Studies have also suggested that the increase of scanning speed up to its centre value improves the polymer weld strength^47,48^. The reduction of laser interaction time is necessary to prevent the shrinkage and deterioration of the welded region. At a higher scanning speed, the laser contact duration decreases, thereby reducing the energy input to the weld zone and prevent the formation of defects. Additionally, heat loss can be minimised by the faster scanning time owing to the reduced time for heat conduction^45,49^. Although the lessened laser polymer interaction time leads to a lowered energy input to the weld region, which in turn reduces the weld shear strength, the multiple scans of the polymer permit the adequate medium power, heat input to be supplied to the SEBS/PP polymer in order for the welding to take place. This shows that it is possible to achieve optimal welding with medium laser power level and higher scanning speed. This implies that at medium laser power coupled with triple scans, optimum melt (molten polymer) volume and weld strength can be achieved despite the higher scanning speed which prevents the formation of defects in the SEBS/PP polymer. The medium laser power efficiently melts the SEBS/PP samples and retains it in a molten state without overheating the polymer during the scan cycle^51^. The temperature at the welding zone increases with the number of scans thereby improving polymer mixing in the melt pool. As a result, the weld strength is increased at the joint interface. Additionally, the increase in the number of scans is accompanied by an increase in weld width and weld depth^51^. Increasing the laser power while maintaining the high scanning speed may result in ablation and material degradation as a result of high heat input that cannot be entirely offset by the high scanning speed. Additionally, a further increase of the scanning speed will significantly decrease the weld strength as the interaction time between the laser and the polymeric material becomes limited^48^. The laser welding study illustrates that it is possible to maximise the weld strength of SEBS/PP through the use of medium laser power requirement and increasing the scanning speed if the number of scans is optimised. This is a compromise process to balance medium laser power and high laser scanning speed.

The great difficulty in welding the SEBS/PP blow-moulded polymers in initial parameters can also be attributed to a significant drawback of laser welding: it usually requires a dark absorptive material for the base layer. The nature of the challenge is exacerbated by the fact that the SEBS/PP is highly transparent, notwithstanding its absorptive capabilities. The level of complexity in laser welding is significantly higher when welding two transmitting (transparent) layers^40,52^. The weldability of the SEBS/PP film is also affected by several factors and chief among them is the crystallinity of the polymers. The semi-crystalline PP has the tendency to scatter light to a greater extent as compared to the amorphous SEBS^53^. This tendency leads to a longer laser path and thus leads to high laser absorption. Essentially, the crystalline structure of PP increases the chances of the laser beam being absorbed or back-scattered. However, when blended with the highly amorphous SEBS, the transmittance of the blend increases, leading to a decrease in laser absorbance and inadvertently reducing the weldability of the blend. Blending a high percentage of SEBS with PP may cause the PP to possess a transmittance equal to amorphous SEBS. While the transmittance may broadly reflect the amorphous SEBS due to its quantity, various crystalline phases in the blend may still affect weldability in other ways^54^. An increase in polymer thickness magnifies crystalline materials' scattering, reflection and absorption effect and reduces transmission. However, this factor does not supersede the effects of CO_2_ laser parameters, as the results show that the samples could not be successfully welded.

### Oxygen permeability test

High oxygen permissibility is essential for blood products, especially for the platelets to maintain aerobic respiration and to be stored for an extended period. Prior research has shown that blood bags with higher oxygen permeability causes less deterioration of blood or blood products and preservation of pH over a period of time^55^. On the other hand, even a minor shortage of oxygen can interfere with aerobic energy generation, thus leading to anaerobic glycolysis which produces acidic conditions and decreases pH, thereby aggravating the total platelet function^55,56^. This implies that high oxygen permeability leads to better storage of blood products. The oxygen transmission rates (OTR) of the samples for all replicates in all aging conditions are provided in Table 3. In this study, the blow-moulded SEBS/PP has a markedly higher oxygen transmission rate than the PVC-based blood bags, indicating the SEBS/PP film has better oxygen permeability. Table 3 shows that the oxygen transmission rate of the commercial samples is within a narrow range without much deviation.

**Table 3:** Oxygen transmission rates (OTR) of commercial PVC-DEHP and blow-moulded SEBS/PP.

Test specimen	OTR Values (cc/m^2^/24 h)											
	Before accelerated aging				20 days of accelerated ageing				40 days of accelerated ageing			
	Number of replicates				Number of replicates				Number of replicates			
	1	2	3	Average	1	2	3	Average	1	2	3	Average
Commercial PVC-DEHP	668.4	528.7	767.1	654.7 ± 119.8	658	561.3	706.9	642.1 ± 74.1	661.2	595.1	731.8	662.7 ± 68.4
Blow-moulded SEBS/PP	1322.1	1369.7	1996.6	1562.8 ± 376.4	1349.4	1428	1436.9	1404.8 ± 48.2	1532.6	1484.7	1442.4	1486.6 ± 45.1

The findings show that there is a significant difference between the oxygen transmission rate of commercial PVC-DEHP and blow-moulded SEBS/PP before and after accelerated aging where the p-value < 0.05.

Moreover, the SEBS/PP samples show a minor but negligible drop-in oxygen transmission rate after 20 and 40 days of accelerated aging. One of the factors that SEBS/PP has a higher oxygen permissibility could be due to its thinner film^55^. It is of significant note that while the SEBS/PP film is slightly thinner than commercial blood bags, the PVC-based bags are larger and supposed to overcome the limitation of thicker size by possessing a larger surface area for gaseous exchange. However, a previous study has shown that certain polyolefins are more permeable to oxygen than PVC and standard PVC blood bags are not maximally oxygenated^57^. This factor permits a higher oxygen concentration to pass through for gaseous exchange of oxygen and carbon dioxide. Incidentally, PVC-based blood bags are primarily utilized for its durability despite the glaring shortcoming of oxygen permeability^31^. The higher weight percentage of amorphous SEBS is possibly another primary factor for the high oxygen transmission rate besides the thickness of the film^58^. This is because the crystalline regions in the semi-crystalline PP are regarded to be impermeable to gas. Hence, by decreasing the crystallinity of the blend as a total, the gas solubility increases owing to the percentage of amorphous SEBS content and increase in diffusion reduction owing to the presence of an easier path for the diffusing gas molecules. The oxygen molecules initially dissolve in the amorphous regions and then penetrate to the depths of the film across the gaps in the amorphous region of the molecular chain, as the gas molecules will not be able to move in the crystallization region.

### Water permeability test

This test pertains to the barrier capabilities of the blood bag. The water vapour transmission rates (WVTR) of the commercial PVC-DEHP and SEBS/PP films are crucial to the fabrication of blood bags as the water permeability of blood bags must be low to prevent water loss from the stored RBCs^24,59^. The WVTR test permits quantifying the amount of water that diffuses through the film per unit of area, time, and pressure gradient (g/s·m·Pa)^60^. The water vapour may pass through polymeric materials by utilizing the pores or cracks in the morphological structure of the polymer and by employing the solubility–diffusion effect due to the combination of Fick’s law of diffusion and Henry’s law of gas solubility^60,61^. The permeability due to the solubility–diffusion effect is described as the actual permeability. The solubility–diffusion model is in which the water molecule is initially transported to the interface of the polymer film and followed by adsorption of the water molecule on the polymer surface in the direction of the region with high concentrations of water vapour. The dissolution of the water vapour molecule on the surface thereafter encourages diffusion in the direction of the driving force upon which the water molecule's desorption occurs on the polymer film's alternate side^60^.

The WVTR of both the samples for all replicates in all aging conditions are provided in Table 4. While the physical aesthetics of the polymers remain largely unchanged, the accelerated aging exhibits minor but negligible changes in the water vapour transmission rates of both polymers. The commercial PVC-DEHP exhibits lower WVTR for both 20 days and 40 days of accelerated aging. Additionally, the WVTR of 20 days and 40 days after accelerated are similar with no or negligible deviation indicating the WVTR is stable with time. The drop in WVTR after accelerated aging is negligible. Despite the higher thickness of the commercial sample, the commercial sample exhibits a higher WVTR indicating a high-water permeability as compared to the SEBS/PP blend polymer which is undesirable and unconducive to storing to the RBCs. Due to its hydrophilic nature, the relatively higher WVTR of the commercial PVC-DEHP can be attributed to the PVC-based blood bag sample's low water barrier properties^20^. The hydrophilic nature of the commercial blood bag may be attributed to the presence of polar groups in its molecular structure. The interactions of these polar groups with the water vapour molecules cause the water vapour permeability to depart from ideal behaviour^62^. This deviation can be ascribed to the variation of the material structure owing to its governance by the free volume theory. Briefly, the water vapour molecules increase the polymer free volume which allows the polymer chain segments to increase its mobility which in turn results in high water vapour permeability. Interestingly, both PVC and DEHP are hydrophobic substances^63,64^. Therefore, there are high possibilities that the hydrophilic nature of commercial blood bags arises from the processing additives incorporated into the blood bags. Despite its permeability, the commercial PVC-DEHP still falls within the permissible range because the polymeric material is expected to absorb water vapour due to its hydrophilicity and thereby contribute to the allowable range of WVTR^58^. Additionally, the higher water vapour permeability of the commercial PVC-DEHP can also be ascribed to high diffusivity due to weak intramolecular and intermolecular attractive forces in the PVC-DEHP polymer matrix^65^.

**Table 4:** Water vapour transmission rates (WVTR) of commercial PVC-DEHP and blow-moulded SEBS/PP.

Test specimen	Meant apparent of water transmission rate (g/h m^2^)											
	Before accelerated aging				20 days of accelerated ageing				40 days of accelerated ageing			
	Number of replicates				Number of replicates				Number of replicates			
	1	2	3	Average	1	2	3	Average	1	2	3	Average
Commercial PVC-DEHP	0.289	0.289	0.253	0.277 ± 0.021	0.184	0.257	0.257	0.233 ± 0.042	0.221	0.221	0.221	0.221 ± 0.000
Blow-moulded SEBS/PP	0.145	0.144	0.217	0.169 ± 0.042	0.037	0.074	0.074	0.062 ± 0.021	0.074	0.147	0.074	0.098 ± 0.042

The findings demonstrate that there is a significant difference between the water transmission rate of commercial PVC-DEHP and blow-moulded SEBS/PP before and after accelerated aging where the p-value < 0.05.

On the other hand, the SEBS/PP polymer, with its relatively higher hydrophobic nature, hinders the free passage of water vapour through its structure. In the case of relatively hydrophobic materials such as the SEBS/PP blend, the water vapour permeability is a proportionality constant that acts independently regardless of the water vapour pressure gradient acting upon the polymer film^62^. The hydrophobicity of the individual polymers such as naturally hydrophobic polypropylene, plays a significant role in improving the water barrier capability of the blended material^58^. Additionally, a previous study has shown that polymer blends with hydrophobic nature and possesses high compatibility between the individual polymer components, showcases better hydrophobicity. Therefore, the high water barrier properties of the SEBS/PP welded films may be attributed to the good interfacial adhesion between SEBS and PP brought about by the segmental diffusion of ethylene–butylene (EB) mid-blocks with PP matrix^66^.

Additionally, the compatibilization of the polymer blends has been demonstrated to reduce voids and free space inside the blend thereby hindering the water molecules from diffusing through the polymer film^58^. The WVTR of the blow-moulded SEBS/PP before accelerated aging is higher than the 20 and 40 days of accelerated aging. This deviation can be ascribed to changes in crystalinity of the sample.

### Shelf life

The durability of synthetic polymers is crucial as the service life, shelf life and storage life of polymeric materials in atmospheric conditions constitute the acceptable lifetime of a medical device^67^. The weatherability and shelf life of blood storage material made of synthetic polymers is highly important. The material needs to withstand external stresses such as oxygen, heat and relative humidity to safely store red blood cells without polymeric material deterioration or device failure^68^. Because no processing additives were added to the SEBS/PP blend to prevent any chemical migration to the surface of the polymer or leach out into the blood, it is necessary to analyze the capability of the blow-moulded SEBS/PP film to withstand atmospheric conditions. The primary visual inspection that was carried out on both test samples consisted of colouration and material transparency inspection. Discolouration or change in colour would be the primary indicator of polymer degradation. In this study, both materials did not exhibit any change in colour despite the accelerated aging process. The small spots that were on the sample surface commercial PVC-DEHP samples were determined to be the presence of anticoagulant residues in the blood bag. The residual material can be wiped away and does not affect the service life of this material. Additionally, the transparency of the test samples is not in any way affected. The SEBS/PP films have a higher transparency than the commercial PVC-DEHP as described in a previous study^20^. It is important to note that the welded samples are relatively thinner compared to the commercial samples. However, this does not affect the transparency of the samples and consequently on its shelf life. Throughout the exposure period, the aging process do cause any alteration of significance. No other significant changes were observed in the aesthetics of both the polymers subjected to shelf-life study. These results indicate that the welded SEBS/PP samples can store the red blood cells for an extended period.

The transparency of blood bag is particularly crucial for visual inspection of the blood bags, and blood and its components in order to assess the viability and functionality of the blood cells as well as to determine the performance of the blood bags during blood storage. The significance of the higher transparency of the SEBS/PP films compared to the commercial PVC-DEHP samples is that that the higher transparency permits improved visual inspection of the blood bags. The visual inspection is required for the assessment of the physical integrity of the blood bags and detection of any physical damage such as leakages or breakages^69^. In addition to that, the higher transparency of SEBS/PP will be able to provide improved visual assessments of blood and its components to check for discernible changes in the physical quality in blood component quality^70,71^. The higher transparency can also be utilised to check for reddish supernatant discoloration which is indicative of haemolysis. The primary visual identification of haemolysis is crucial to validate the quality blood products during its storage life for safe transfusion. Free haemoglobin accumulates in the stored blood when blood is haemolysed and leads to storage lesions that affects the patients’ response to blood transfusion. The assessment of the stored blood throughout the storage life is crucial as haemolysis may occur in storage unit due to reasons such as thermal injury during blood collection, transportation, preservation and or different stages of handling in the blood bank^72^. Furthermore, if SEBS/PP polymers are used for the storage of plasma, the higher transparency would allow for better visual checks for the presence of opacities (fibrin) and discoloration whereas if it is used for the storage of platelets, the transparency can be used for the improved visual determination of platelet clumps and opacities (fibrin)^70^. Moreover, using SEBS/PP for plasma bags will permit heightened visual inspection of turbidity caused by increased levels of triglycerides thus allowing for the disposal of plasma in order to mitigate the risk of transfusion^73^.

Other than that, the high visual inspection capability of SEBS/PP can also be utilised for the detection of bacterial contaminants in blood as bacterial contamination of donated blood and its components is a major issue during blood storage^74,75^. Primarily, the contaminating bacteria consists of coagulase-negative *Staphylococci*, *Bacillus* spp., *Escherichia coli*, *Klebsiella* spp., *Streptococci* spp., *Enterobacter* spp., and *Citrobacter* spp., and *Staphylococcus aureus* which may be introduced into the blood endogenously (from the donor) or exogenously (during collection and processing)^74,76^. The bacterial contamination of blood can be viewed in the blood bag by checking for the presence of unnecessary and unusual air bubbles which forms a pink to red discoloration that is visible in the supernatant. Bacterial contamination of blood and its components can cause severe morbidity or even mortality during transfusion therapy and as such the pre-release visual inspection of blood and its components is highly important. Furthermore, SEBS/PP also allows for better inspection of any visible changes such as clumping, abnormal volume, particulate matter, lipemia, red blood cell contamination, icterus and any abnormal colour change^77^. To these ends, the higher transparency of the SEBS/PP sample as compared to PVC-DEHP throughout its shelf-life is highly pertinent for the safe storage and transfusion of blood. Visual inspection through the relatively less transparent PVC-DEHP may not permit the optimal requirement for discerning any abnormalities that may not be readily visible. A visual inspection of the blood bag is required throughout the storage life of the blood unit as it is not known whether the blood unit will be optimal throughout its storage life^78,79^. A proper visual inspection of the bag at varying intervals may reveal blood which appears whole and healthy but may possess any ill effects during the storage life of the blood unit.

### Bioburden test

In order to determine the bioburden estimates of both the SEBS/PP and PVC-DEHP samples, the correction factor of both samples was determined. Table 5 shows the results of the bioburden tests with the standard deviations of the number recovery by 1st treatment provided. The average recovery by 1st treatment of the 10 commercial PVC-DEHP replicates is 14%, giving a correction value of 7.1 (Correction value: 100/14 = 7.14). The average number of recovery (CFU) of PVC-DEHP samples is 30 with a maximum of 44 and a minimum of 9. The bioburden estimate of the commercial PVC-DEHP sample is therefore 213 CFU, following the calculation 30 × 7.1 = 213. On the other hand, the average recovery by 1st treatment of the 10 replicates of the blow-moulded SEBS/PP 14.7% which provides a correction value of 6.8, from the calculation 100/14.7 = 6.8. The average total number recovery (CFU) of blow-moulded SEBS/PP samples is 16.9 with a maximum of 44 and a minimum of 4. Therefore, the bioburden estimate of the blow-moulded SEBS/PP is 115, following the calculation 16.9 × 6.8 = 114.9. It is clear from these results that the bioburden estimates of both the commercial PVC-DEHP and blow-moulded SEBS/PP are lower than 1000 CFUs implying that the repetitive recovery method can be suitably applied for these samples. However, the PVC-DEHP sample generally has a higher bioburden than the SEBS/PP test sample. Additionally, the SEBS/PP sample has a bioburden CFU value below a factor of 2, implying that the SEBS/PP sample has an acceptable CFU count between 50 and 200 CFU as specified in United States Pharmacopeia-61 (USP 61)^80,81^. However, the bioburden estimate for the commercial PVC-DEHP slightly exceeds the acceptable value, thus indicating that SEBS/PP is more suitable as blood bag polymer material.

**Table 5:** Bioburden test of commercial PVC-DEHP and blow-moulded SEBS/PP.

Test specimen	Replicates	1	2	3	4	5	6	7	8	9	10	Average
Commercial PVC-DEHP	Number recovery by 1st treatment	4	3	5	1	2	7	1	5	6	1	3.5 ± 2.2
	Total number recovery (CFU)	35	44	13	21	30	32	9	19	59	38	30 ± 15.1
	Recovery by 1st treatment (%)	11.4	6.8	38.5	4.8	6.6	21.9	11.1	26.3	10.2	2.6	14 ± 11.4
Blow-moulded SEBS/PP	Number recovery by 1st treatment	2	8	2	2	1	2	2	2	0	1	2.2 ± 2.1
	Total number recovery (CFU)	15	44	13	17	11	16	36	4	4	9	16.9 ± 13.1
	Recovery by 1st treatment (%)	13.3	18.2	15.4	11.8	9.1	12.5	5.6	50	0	11.1	14.7 ± 13.4

The results showcase that there are no significant differences for both commercial PVC-DEHP and blow-moulded SEBS/PP as the p-value > 0.05.

There are a number of factors that can be attributed as the causes of the relatively higher bioburden levels in PVC-DEHP as compared to the SEBS/PP polymer. The surface roughness of the tested samples plays a crucial role in determining the number of microbes on the surface of the polymers. PVC-DEHP which has a higher surface roughness can be attributed as a cause for the higher bioburden estimate as an increase in surface roughness is commonly accompanied by an increase in microbial adhesion and biofilm formation^82^. This is due to the fact that polymers with higher surface roughness such as PVC-DEHP increases the surface area available for microbial attachment and acts as a scaffold for adhesion. In addition to that, rough surfaces have the tendency to resist the detachment of bound microbes as the rough surface protects the microbes against shear forces. Moreover, the lower bioburden of SEBS/PP samples can be attributed to the adhesion resistance of the hydrophobic surface owing to the material’s lower surface energy^83^. The low energy surface diminishes the number of microbial adhesions as the surface becomes more accessible to microbial sloughing and cleaning due to the weaker binding forces at the interface.

Despite the fact that microbes have a tendency to adhere to hydrophobic surfaces as opposed to hydrophilic surfaces as elucidated by Saeki et al. (2016) and Yuan et al. (2017), the bioburden estimate of the SEBS/PP sample is lower than that of PVC-DEHP^84,85^. However, studies have shown that hydrophobic surfaces are capable of discouraging microbe attachment through the optimal balance of hydrophobicity and other physicochemical surface properties^28,86,87^. The lower bioburden on SEBS/PP can also be attributed to the interactions such as electrostatic forces acting in conjunction with low surface energy and surface smoothness to deter the attachment of microbes of the SEBS/PP surface^88,89^. As medical devices such as blood bags has the potential to be contaminated by microorganisms via high touch surfaces potentially leading to infections, it is crucial that the potential of blood bags to carry microbial loads be reduced^90^. This factor is further exacerbated by the fact that hospital settings where medical devices are more prone to contamination. The lower bioburden of SEBS/PP samples is in line with current efforts to fabricate materials with surfaces that reduce or inhibit the capability of microbes as a lower bioburden implies easier sterilization. On the other hand, the use of PVC-DEHP based blood bags are sub-optimal as it carries the risk of having a higher bioburden as compared to SEBS/PP polymers. This need for a lower bioburden is often hampered by product limitations and perceived risks. As such, SEBS/PP polymer which has a lower bioburden without the associated risks is necessary.

## Conclusion

Our research reveals conclusively that the SEBS/PP prototype has more performance capabilities than standard PVC blood bags. This research enhances our prior investigation and strengthens the rationale for employing SEBS/PP as blood bags. The results indicated that the optimal CO_2_ laser welding parameters to obtain adequate sealing are 60% (power), 70 in/sec (speed) and 500 (PPI) with triple laser scans. This study concludes that it is feasible to laser weld transparent SEBS/PP polymer layers at medium power with high scanning speed provided that multi-pass scanning is conducted to achieve adequate melting and mixing of SEBS/PP polymer. Stronger clamping force to increase seal strength during laser welding in order to improve the thermal contact conduction between the two polymeric layers may be used if necessary. On the other hand, the high oxygen permeability rate of 1486.6 cc/m^2^/24 h allows for better RBC respiration and may even be used for storing blood components such as platelets considering its high oxygen requirement. The oxygen permeability of SEBS is slightly more than double the oxygen permeability of PVC-DEHP, which allows adequate gas exchange and prevents CO_2_ buildup that can lead to increase in stored blood pH. Additionally, the observed lower water transmission rate of SEBS/PP polymer of 0.098 g/h.m^2^ after 40 days of ageing is of particular interest as high water barrier properties will hinder the evaporation of water during blood storage. Furthermore, the blow-moulded SEBS/PP has a lower bioburden estimate of 115 CFU as compared to the commercial PVC-DEHP which has a bioburden estimate of 213 CFU. The lower bioburden estimate in SEBS/PP can be attributed to its smooth surface and low surface energy that has a better tendency to resist microbe attachment as compared to PVC-DEHP. In light of these factors, the authors foresee the potential of using SEBS/PP-based based bags for critical medical applications, provided that the storage time and quality of the RBCs are not adversely affected.

## Data Availability

The data presented in this study are available on request from the corresponding author.
